# NLoS Mitigation with Propagation Reliability Estimation for Track-Constrained UWB/IMU Fusion Localization

**DOI:** 10.3390/s26154680

**Published:** 2026-07-23

**Authors:** Run-Ze Tan, Jin-Feng Chen, Wan-Ning He

**Affiliations:** 1 Department of Communication Engineering, Tongji University, Shanghai 200092, China; 2351874@tongji.edu.cn; 2 Department of Information and Communication Engineering, Tongji University, Shanghai 200092, China; 2410943@tongji.edu.cn

**Keywords:** ultra-wideband (UWB), inertial measurement unit (IMU), rail transportation localization, track-constrained localization, LoS/NLoS interference, NLoS mitigation, propagation reliability estimation

## Abstract

Ultra-wideband (UWB) and inertial measurement unit (IMU) fusion is an effective scheme for train and rail-guided target localization because UWB provides absolute ranging results and the IMU provides high-rate motion prediction. In practical rail transportation systems, UWB anchors are usually deployed along the track with large longitudinal intervals and limited lateral separation to reduce installation and maintenance costs. This anchor deployment leads to a large condition number of the observation matrix, so slight ranging errors caused by non-line-of-sight (NLoS) propagation may be amplified into large localization errors. To mitigate LoS/NLoS interference, this article proposes a propagation reliability estimation method for track-constrained UWB/IMU fusion localization. First, rail transportation localization along a narrow path is formulated as a one-dimensional track-constrained problem, and each UWB ranging result is converted into a candidate longitudinal coordinate on the known track centerline. Second, the reliability of each anchor–target propagation is estimated in a sliding window by comparing the motion increments solved by UWB observations with the motion prediction by the IMU. Third, the estimated reliability is incorporated into a reliability-weighted track-domain update before a closed-loop position–velocity Kalman correction. The simulation results show that, under the mixed LoS/NLoS scenario, the proposed method achieves an NLoS-interval RMSE of 0.0090 m. Compared with Track-EKF, Track-Gauss-AUKF, Track-Adaptive KF, and Track-SW-FGO, the proposed method reduces the NLoS-interval RMSE by 92.9%, 62.1%, 92.8%, and 92.6%. A supplementary real-data stress test on the public STAR-loc dataset demonstrates an average longitudinal RMSE of 0.0555 m under a strict online calibrated-range protocol, supporting the algorithm’s practical applicability against real-world lateral sway and asynchronous sensor noise.

## 1. Introduction

Reliable localization is essential for rail transportation systems and track-constrained automated guided vehicles (AGVs). In these applications, a mobile target moves along a predefined track or guideway and requires continuous position information for safe operation, motion control, and system management. Representative scenarios include railways, subway tunnels, underground mines, and industrial guideways, where global navigation satellite system (GNSS) signals may be unavailable or unreliable.

Several localization solutions are available for rail-guided or track-constrained systems. Wheel- or encoder-based odometry provides continuous relative motion information, but its accuracy may degrade because of cumulative integration errors, wheel slip, wheel wear, and calibration uncertainty [1]. RFID tags or balise-like trackside markers can provide absolute position references at known locations, although their corrections are discrete and depend on the deployment density of the trackside infrastructure [2]. LiDAR map matching and multi-modal SLAM can achieve high localization accuracy in structured rail or tunnel environments, but they generally require additional sensing hardware, high-quality maps, sufficiently distinctive geometric features, and nontrivial computational resources [3,4]. These technologies therefore address different infrastructure conditions and operational requirements.

For applications requiring continuous localization without dense trackside markers or consistently distinctive map features, ultra-wideband (UWB) and inertial measurement unit (IMU) fusion provides a practical complementary solution. UWB provides absolute ranging observations through time of arrival (ToA), whereas an IMU provides continuous and high-rate short-term motion prediction. Their complementary characteristics have motivated UWB/IMU localization in rail transportation, tunnel, underground-mine, and other GNSS-denied narrow-path environments [5,6,7,8,9,10].

**Figure 1:**
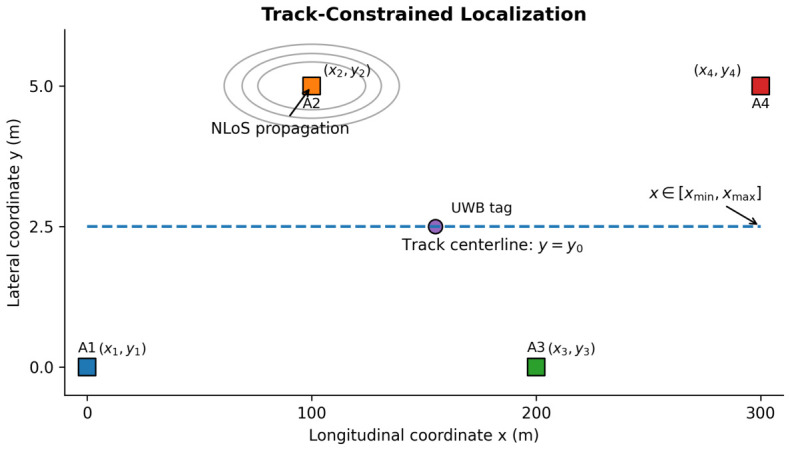
Track-constrained narrow-path scenario.

UWB anchor deployment in rail-guided environments differs from that in general indoor localization. To reduce installation and maintenance requirements, anchors are commonly distributed along the track with large longitudinal intervals and limited lateral separation, as shown in Figure 1. Such a narrow-path deployment may produce an ill-conditioned observation geometry, under which small ranging errors are amplified into large localization errors [6,11,12]. The problem becomes more severe under non-line-of-sight (NLoS) propagation caused by tunnels, power poles, platform structures, train bodies, vehicles, passengers, or industrial equipment. Reliable track-constrained localization therefore requires both continuous UWB/IMU fusion and effective suppression of unreliable UWB propagations.

The existing UWB NLoS mitigation studies broadly follow two directions: (1) directly reducing the influence of abnormal ranging observations through robust estimation, convex formulations, or outlier-resistant optimization; and (2) identifying LoS/NLoS conditions and subsequently correcting, suppressing, or selecting measurements using channel statistics, ranging-error models, or data-driven rules [13,14,15,16,17,18]. Meanwhile, Kalman-filter variants, adaptive filtering, smoothing, and factor-graph methods have been widely used for UWB/IMU fusion [9,19,20,21,22,23,24,25]. These methods improve overall robustness, while most use IMU measurements primarily as process inputs and UWB ranges as measurement updates. They therefore lack an explicit mechanism for evaluating the reliability of each anchor–target propagation from its consistency with the predicted target motion before the final state update.

A key gap consequently remains in track-constrained UWB/IMU localization. The track constraint reduces the feasible state space and removes the weak lateral mode, but it does not determine which UWB propagation should be trusted under time-varying LoS/NLoS interference. Under ill-conditioned narrow-path geometry, even one unreliable propagation may dominate the longitudinal update. This motivates a propagation-level reliability mechanism that uses IMU-predicted motion as a temporal consistency reference while retaining useful information from reliable UWB observations.

To address this gap, this article proposes an NLoS mitigation method with motion-consistency-based propagation reliability estimation for track-constrained UWB/IMU fusion localization. Each UWB range is first converted into a candidate longitudinal coordinate on the known track centerline. The longitudinal motion increment inferred from each anchor–target propagation is then compared with the short-term motion increment predicted by the IMU over a sliding window. The resulting soft reliability weights are incorporated into a reliability-weighted track-domain update, while excess-range bias compensation, residual gating, hold suppression, and closed-loop position–velocity Kalman correction improve stability under abrupt and persistent NLoS interference.

The main contributions of this article are summarized as follows:

1. **Track-domain UWB/IMU fusion formulation.** We formulate rail-guided localization as a one-dimensional track-constrained estimation problem. Each UWB range is converted into a candidate longitudinal coordinate on the known centerline, enabling per-anchor motion-consistency evaluation in a unified track domain.
2. **Motion-consistency-based propagation reliability estimation.** We estimate the reliability of each anchor–target propagation by comparing UWB-derived longitudinal motion increments with IMU-predicted short-term motion increments in a sliding window. This provides a soft propagation-level reliability measure before the final localization update.
3. **Reliability-weighted NLoS mitigation with closed-loop correction.** We incorporate the estimated propagation reliability into a bias-augmented track-domain update with residual gating and hold suppression. A closed-loop position–velocity Kalman correction then suppresses LoS/NLoS interference while reducing open-loop inertial drift.

Table 1 lists the principal abbreviations used throughout the article.

The remainder of this article is organized as follows. Section 2 reviews related localization methods. Section 3 describes the track-constrained ToA model and analyzes the effect of ill-conditioning of anchor deployment. Section 4 presents the proposed propagation-reliability-weighted fusion scheme. Section 5 explains the design rationale and computational complexity. Section 6 presents the simulation settings and fair track-domain baselines. Section 7 reports the experimental results. Section 8 concludes this article and outlines future work.

## 2. Related Work

This section reviews UWB LoS/NLoS mitigation, UWB/IMU fusion, and track-constrained localization in narrow-path scenarios, with an emphasis on propagation reliability derived from short-term motion consistency.

### 2.1. UWB ToA Localization and LoS/NLoS Mitigation

LoS/NLoS interference is a central issue in ToA-based UWB localization. One line of studies mitigates ranging errors caused by NLoS propagation directly through convex relaxation, robust estimation, or message passing. For example, convex optimization and iterative message-passing methods reduce the effect of unreliable ranging results in snapshot localization [13,14]. Robust cooperative localization and outlier-resistant estimation have also been studied to reduce the influence of measurement outliers [15]. Another line of studies first identifies LoS/NLoS propagations and then corrects, suppresses, or selects ranging results by using channel statistics, anchor selection, or learning-based rules [12,16,18]. These methods improve ranging quality or snapshot localization accuracy at the UWB-measurement level. Rail-guided motion constraints and IMU-predicted short-term motion are generally outside their measurement models.

### 2.2. Fusion Localization Based on IMU and UWB

An IMU and UWB are complementary sensors for continuous localization. An IMU provides high-rate motion prediction, whereas UWB provides absolute ranging correction. Therefore, KF, EKF, adaptive KF, UKF, and factor-graph methods have been widely studied for UWB/IMU fusion localization [9,10,20,21,22,23]. These methods usually use IMU measurements as process inputs and UWB ranging results as measurement updates. When LoS/NLoS interference appears, they often adapt the covariance or apply robust loss functions. This strategy can reduce the global influence of unreliable measurements, but it does not directly identify which anchor–target propagation should be trusted at each time. In addition, robust loss functions may respond slowly when NLoS propagation changes gradually.

### 2.3. Track-Constrained Localization Along a Narrow Path

Rail transportation localization is a typical narrow-path problem. UWB anchors are often deployed along the track with large longitudinal intervals and limited lateral separation. The observation matrix can therefore have a large condition number, so slight ranging errors may cause large localization errors. The narrow-path benchmark in [6] systematically analyzed this issue under LoS/NLoS interference. Related railway and map-assisted studies have shown that anchor deployment, Doppler effects, subway ranging missingness, and map constraints can affect UWB/IMU or UWB/INS localization performance [7,8,11,26]. These studies confirm the value of track or map information. A track constraint limits the feasible state space, while propagation reliability under time-varying LoS/NLoS interference remains unresolved.

### 2.4. Research Gap

The reviewed literature addresses measurement-level NLoS suppression, state-level UWB/IMU fusion, and map constraints through complementary approaches. A remaining issue in narrow-path deployment is the absence of a propagation-specific temporal consistency variable before state correction. The proposed method constructs this variable by comparing UWB-derived longitudinal motion increments with IMU-predicted short-term motion increments in a sliding window and evaluates the resulting estimator using consistent one-dimensional track-domain baselines.

## 3. System Model and Problem Formulation

### 3.1. Track Constraint in Narrow-Path Scenarios

We consider a two-dimensional horizontal plane with *M* UWB anchors at known positions $s_{i}={\left[x_{i},y_{i}\right]}^{⊤}$ and a mobile target at $p={\left[x,y\right]}^{⊤}$. The general track centerline can be represented as a known parametric curve $c\left(s\right)={\left[c_{x}\left(s\right),c_{y}\left(s\right)\right]}^{⊤}$, $s\in [s_{0},s_{1}]$. The experiments in this article use the straight-track special case

(1) $$T=\{\left(x,y\right)\in R^{2}|x\in \left[x_{\min},x_{\max}\right],\;y=y_{0}\}.$$

The set T contains all feasible target positions on the straight track. Here, $x_{\min}$ and $x_{\max}$ are the lower and upper longitudinal limits of the track, and $y_{0}$ is the known lateral coordinate of the track centerline. The target state is therefore reduced from ${\left[x,y\right]}^{⊤}$ to the scalar longitudinal coordinate *x*. Figure 1 shows the narrow-path scenario, in which anchors are longitudinally separated but have limited lateral spread.

The hard constraint $y=y_{0}$ represents nominal centerline motion for a rail-guided target. Mechanical guidance limits lateral sway relative to free two-dimensional motion, while residual sway and centerline-map error enter the model as mismatch. A dedicated centerline-offset sensitivity analysis is presented later in Section 7.6.

The hard centerline constraint imposes a direct accuracy requirement on the track map. Since the estimator outputs ${\hat{p}}_{k}={\left[{\hat{x}}_{k},y_{0}\right]}^{⊤}$, an offset between the assumed and true motion centerlines appears as a systematic localization error. If the lateral centerline error is denoted by $\Delta y_{c}$, the two-dimensional localization error contains at least the component $|\Delta y_{c}|$ even when the longitudinal coordinate is accurately estimated. Therefore, for an application with a required two-dimensional accuracy $e_{\mathrm{req}}$, the centerline error and the allowed lateral sway should be included in the error budget. A practical requirement is

(2) $$|\Delta y_{c}{|}_{\mathrm{rms}}+e_{\mathrm{sway},\mathrm{rms}}\ll e_{\mathrm{req}},$$

and both terms should remain below the required positioning accuracy. This requirement targets rail-guided, tunnel-guided, and AGV systems whose centerline can be surveyed or calibrated to the same order of accuracy as the desired localization performance.

### 3.2. UWB Ranging Model Under LoS/NLoS Interference

For a target on the straight centerline, the predicted ranging distance from anchor *i* is

(3) $$r_{i}\left(x\right)=\sqrt{{\left(x-x_{i}\right)}^{2}+{\left(y_{0}-y_{i}\right)}^{2}}.$$

The measured UWB ranging result is written as

(4) $$d_{k,i}=r_{i}\left(x_{k}\right)+\epsilon_{k,i}+z_{k,i}b_{k,i},$$

where $\epsilon_{k,i}∼N\left(0,\sigma_{\mathrm{LoS}}^{2}\right)$ is the zero-mean LoS ranging noise, $z_{k,i}\in \left\{0,1\right\}$ is the NLoS indicator, and $b_{k,i}\geq 0$ is the ranging error caused by NLoS propagation. The original benchmark uses a Gaussian ranging-error setting, $b_{k,i}∼N^{+}\left(\mu ,\sigma_{\mathrm{NLoS}}^{2}\right)$, where $N^{+}$ denotes truncation to non-negative values [6]. Later experiments also use exponential, lognormal, Gaussian-mixture, AR(1), and correlated blockage models affecting multiple propagations to evaluate robustness across NLoS bias distributions, temporal correlations, and simultaneous blockage of multiple propagations.

### 3.3. Impact of Narrow-Path Anchor Deployment on ToA Localization

For an unconstrained two-dimensional target, the ranging Jacobian matrix is

(5) $$J\left(p\right)=\frac{\partial {\left[r_{1}\left(p\right),\ldots ,r_{M}\left(p\right)\right]}^{⊤}}{\partial [x,y]}=\left[\begin{array}{cc} \frac{x-x_{1}}{r_{1}\left(p\right)} & \frac{y-y_{1}}{r_{1}\left(p\right)}\\ \vdots & \vdots \\ \frac{x-x_{M}}{r_{M}\left(p\right)} & \frac{y-y_{M}}{r_{M}\left(p\right)} \end{array}\right].$$

Let δp be a small perturbation of the target position and let δd be the corresponding perturbation of the ideal ranging vector. The vector n denotes the ranging-error vector caused by LoS noise and possible NLoS propagation. Linearizing the ranging model gives

(6) δd=J(p)δp+n,

where higher-order terms are ignored. If J has full column rank, the least-squares perturbation induced by n satisfies

(7) $$\delta \hat{p}={\left(J^{⊤}J\right)}^{-1}J^{⊤}n=J^{†}n,\;{∥\delta \hat{p}∥}_{2}\leq \frac{1}{\sigma_{\min}\left(J\right)}{∥n∥}_{2}.$$

Here, $J^{†}={\left(J^{⊤}J\right)}^{-1}J^{⊤}$ is the Moore–Penrose pseudoinverse for the full-column-rank case, and $\sigma_{\min}\left(J\right)$ is the smallest singular value of J. Thus, when $\sigma_{\min}\left(J\right)$ is small, slight ranging interference can produce large localization errors. The condition number

(8) $$\kappa \left(J^{⊤}J\right)=\frac{\sigma_{\max}^{2}\left(J\right)}{\sigma_{\min}^{2}\left(J\right)}$$

quantifies this amplification. A large $\kappa (J^{⊤}J)$ indicates that the normal matrix is ill-conditioned and that ranging errors are strongly amplified in the two-dimensional localization result. Narrow-path anchor layouts reduce lateral diversity, make the columns of J nearly dependent, and shrink $\sigma_{\min}\left(J\right)$.

The track constraint changes the update from a two-dimensional problem to a scalar problem. Along the centerline, the one-dimensional derivative is

(9) $$j_{x}\left(x\right)={\left[\frac{\partial r_{1}}{\partial x},\ldots ,\frac{\partial r_{M}}{\partial x}\right]}^{⊤},\;\frac{\partial r_{i}}{\partial x}=\frac{x-x_{i}}{r_{i}\left(x\right)}.$$

**Figure 2:**
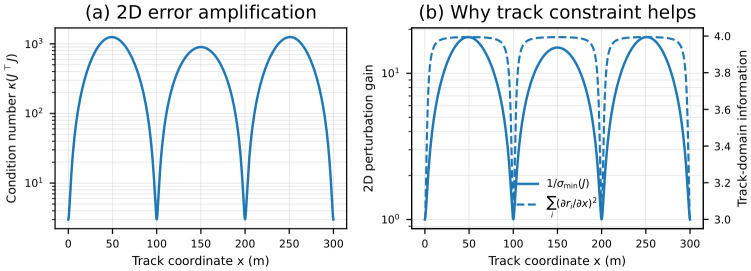
Ill-conditioning mechanism.

The scalar normal information is $j_{x}^{⊤}j_{x}=\sum_{i}{\left(\partial r_{i}/\partial x\right)}^{2}$. This scalar quantity describes how much the UWB ranging results constrain the longitudinal track coordinate. Although it can vary along the track, the poorly observed lateral mode is removed by the track constraint. Figure 2 visualizes both effects: the two-dimensional condition number peaks above $10^{3}$, while the track-domain information remains a scalar longitudinal quantity.

### 3.4. Evaluation Metrics

Given estimates $\left\{{\hat{p}}_{k}\right\}$ and ground truth $\left\{p_{k}\right\}$, the error is $e_{k}={∥{\hat{p}}_{k}-p_{k}∥}_{2}$. We report

(10) $$\mathrm{RMSE}=\sqrt{\frac{1}{N}\sum_{k=1}^{N}e_{k}^{2}},\;P95=Q_{0.95}\left(\left\{e_{k}\right\}\right),\;e_{\max}=\max_{k}e_{k},$$

where *N* is the number of samples, $Q_{0.95}\left(\cdot \right)$ is the 95th-percentile operator, and $e_{\max}$ is the maximum localization error. The failure rate is $\mathrm{Pr}(e_{k}>e_{\mathrm{fail}})$, where $e_{\mathrm{fail}}$ is the failure threshold specified in the experiments.

## 4. Proposed Track-Constrained UWB/IMU Fusion Scheme

**Figure 3:**
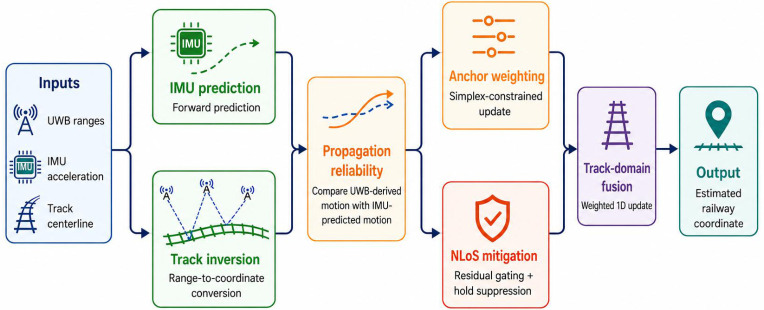
Proposed localization framework.

The proposed framework centers on motion-consistency-based propagation reliability estimation, which extracts a soft reliability measure for each anchor–target propagation from incremental motion consistency before the absolute state update. Track-domain inversion provides comparable UWB-derived motion increments, while residual gating, hold suppression, excess-range bias compensation, and closed-loop Kalman correction stabilize the online update under abrupt and persistent NLoS interference. At each time index *k*, the UWB ranges, the longitudinal IMU acceleration, and the known track centerline are processed in four stages. First, the IMU prediction provides a short-term motion reference, and each UWB range is converted into a candidate longitudinal coordinate on the track. Second, per-anchor motion-consistency evaluation compares UWB-derived longitudinal increments with IMU-predicted motion increments in a sliding window. Third, the estimated propagation reliability is incorporated into a bias-augmented track-domain update with residual gating and hold suppression. Finally, the resulting scalar track-domain estimate is used as a pseudo-measurement to correct both position and velocity through a closed-loop Kalman update. Figure 3 summarizes the overall pipeline.

### 4.1. Closed-Loop IMU Prediction and Per-Anchor Track Inversion

The longitudinal kinematic state is expanded to

(11) $$X_{k}={\left[\begin{array}{cc} x_{k} & v_{k} \end{array}\right]}^{⊤},\;H=\left[\begin{array}{cc} 1 & 0 \end{array}\right],\;E_{v}=\left[\begin{array}{cc} 0 & 1 \end{array}\right],$$

where $x_{k}$ is the longitudinal coordinate, $v_{k}$ is the longitudinal velocity, H extracts position, and $E_{v}$ extracts velocity. The prior state and covariance are propagated by

(12) $$\begin{array}{cc} X_{k|k-1} & =FX_{k-1|k-1}+Gu_{k},\;P_{k|k-1}=FP_{k-1|k-1}F^{⊤}+Q,\\ F & =\left[\begin{array}{cc} 1 & \Delta t\\ 0 & 1 \end{array}\right],\;G=\left[\begin{array}{c} \frac{1}{2}\Delta t^{2}\\ \Delta t \end{array}\right],\;Q=\mathrm{diag}\left(q_{x},q_{v}\right),\\ x_{k}^{\mathrm{pred}} & =\Pi_{\left[x_{\min},x_{\max}\right]}\left(HX_{k|k-1}\right),\;v_{k}^{\mathrm{pred}}=E_{v}X_{k|k-1}. \end{array}$$

Here, $P_{k|k-1}$ is the prior error covariance, Q is the process-noise covariance, $q_{x}$ and $q_{v}$ are the position and velocity process-noise intensities, and $\Pi_{\left[x_{\min},x_{\max}\right]}\left(\cdot \right)$ projects a scalar value onto the valid track interval. Unlike a pure inertial integrator, $X_{k|k-1}$ is corrected after the UWB track-domain update in Equation (27); hence, both $x_{k}$ and $v_{k}$ are in a closed loop.

Each UWB ranging result is converted into a candidate longitudinal coordinate by a bounded one-dimensional residual minimization rather than by truncating a negative square-root argument:

(13) $$x_{k,i}^{\mathrm{inv}}=\underset{x\in [x_{\min},x_{\max}]}{\mathrm{arg min}}\left|\sqrt{{\left(x-x_{i}\right)}^{2}+{\left(y_{0}-y_{i}\right)}^{2}}-d_{k,i}\right|.$$

For the straight-track case, Equation (13) can be solved analytically by checking a small candidate set. Let $\Delta y_{i}=y_{0}-y_{i}$. If $d_{k,i}\geq \left|\Delta y_{i}\right|$, the unconstrained minimizers are $x_{i}\pm \sqrt{d_{k,i}^{2}-\Delta y_{i}^{2}}$; otherwise, the closest feasible range occurs at the anchor projection $x_{i}$. After projection onto $\left[x_{\min},x_{\max}\right]$, ties are resolved by choosing the candidate closest to $x_{k}^{\mathrm{pred}}$. This form preserves the original range residual and avoids the discontinuity introduced by replacing $d_{k,i}^{2}-\Delta y_{i}^{2}$ with $\max(d_{k,i}^{2}-\Delta y_{i}^{2},0)$ inside the model.

### 4.2. Motion-Consistency-Based Propagation Reliability Estimation

Propagation reliability quantifies the temporal consistency of each anchor–target propagation before the final track-domain update. The track-domain formulation converts every UWB range into a candidate longitudinal coordinate, whose temporal variation represents the motion implied by that propagation. Reliable propagation produces a UWB-derived longitudinal increment consistent with the short-term motion predicted by the IMU.

Let

(14) $$\delta_{t}^{\mathrm{imu}}=x_{t}^{\mathrm{pred}}-x_{t-1}^{\mathrm{pred}},\;\ell_{t,i}=x_{t,i}^{\mathrm{inv}}-x_{t-1,i}^{\mathrm{inv}},$$

where $\delta_{t}^{\mathrm{imu}}$ is the short-term motion increment predicted by IMU and $\ell_{t,i}$ is the UWB-derived motion increment from anchor *i*. A reliable anchor–target propagation should generate increments consistent with $\delta_{t}^{\mathrm{imu}}$. Because the LoS/NLoS propagation state usually changes gradually and because a single anchor can be geometrically ambiguous on a line, two regularizers are introduced before estimating the weights: $\lambda_{w}$ penalizes temporal weight chattering, while β discourages collapse to one anchor unless the data strongly support it.

Over the window $W_{k}=\left\{k-N_{w}+1,\ldots ,k\right\}$, propagation reliability weights are obtained by

(15) $$\begin{array}{cc} \min_{\left\{w_{t}\right\}}\;\; & \sum_{t\in W_{k}}{\left(\delta_{t}^{\mathrm{imu}}-w_{t}^{⊤}\ell_{t}\right)}^{2}+\lambda_{w}\sum_{t\in W_{k}}{∥w_{t}-w_{t-1}∥}_{2}^{2}+\beta \sum_{t\in W_{k}}{∥w_{t}-w_{\mathrm{uni}}∥}_{2}^{2},\\ s.t.\;\; & w_{t}⪰0,\;1^{⊤}w_{t}=1,\;\forall t\in W_{k}, \end{array}$$

where $\ell_{t}={\left[\ell_{t,1},\ldots ,\ell_{t,M}\right]}^{⊤}$ collects the UWB-derived increments, $w_{t}={\left[w_{t,1},\ldots ,w_{t,M}\right]}^{⊤}$ is the relative propagation reliability vector, and $w_{\mathrm{uni}}=1/M$ is the uniform vector. With fixed increments, Equation (15) is a convex quadratic program over a Cartesian product of simplexes. Its gradient for an interior window point is

(16) $$\begin{array}{cc} \nabla_{w_{t}}L\; & =-2\ell_{t}\left(\delta_{t}^{\mathrm{imu}}-w_{t}^{⊤}\ell_{t}\right)+2\lambda_{w}\left(w_{t}-w_{t-1}\right)+2\lambda_{w}\left(w_{t}-w_{t+1}\right)+2\beta \left(w_{t}-w_{\mathrm{uni}}\right), \end{array}$$

with boundary terms omitted at the first and last window samples. The term with $w_{t-1}$ comes from the smoothness penalty at time *t*, while the term with $w_{t+1}$ comes from the smoothness penalty at time t+1. We solve Equation (15) by projected gradient descent (PGD). PGD converges to a global optimum of this convex problem for a step size below the inverse Lipschitz constant of the gradient. In practice, the number of anchors is small, and a fixed iteration budget gives deterministic online cost.

The simplex projection follows the standard sorting-based projection. For a vector q, sort its components in descending order $q_{\left(1\right)}\geq \ldots \geq q_{\left(M\right)}$, find

(17) $$\rho =\max\left\{j:q_{\left(j\right)}-\frac{1}{j}\left(\sum_{r=1}^{j}q_{\left(r\right)}-1\right)>0\right\},\;\theta =\frac{1}{\rho }\left(\sum_{r=1}^{\rho }q_{\left(r\right)}-1\right),$$

and set

(18) $${\left[\Pi_{\Delta }\left(q\right)\right]}_{i}=\max\left(q_{i}-\theta ,0\right).$$

This projection enforces both non-negativity and unit-sum normalization, so the resulting weights remain interpretable as relative propagation reliability.

The optimization yields soft propagation-level weights. Snapshot residual-threshold methods operate on instantaneous residuals, whereas Equation (15) evaluates temporal motion consistency over a sliding window. Adaptive covariance filtering changes the confidence of the overall measurement update through covariance inflation; the proposed estimator instead assigns separate weights to the anchor–target propagations before state correction. The resulting update suppresses unreliable propagations while retaining active LoS information.

### 4.3. Bias-Augmented Gating, Hold Suppression, and Closed-Loop Track Update

First differencing attenuates a nearly constant NLoS excess range even though the same bias still corrupts the absolute range update. The final track-domain update therefore estimates a propagation-specific non-negative excess-range bias, while residual gating controls abrupt range changes before the update. The pre-gating prediction residual of anchor *i* is

(19) $$\rho_{k,i}=\left|d_{k,i}-r_{i}\left(x_{k}^{\mathrm{pred}}\right)\right|,$$

where $\rho_{k,i}$ measures the mismatch between the measured range and the range predicted from the prior track coordinate. The robust aggregate residual is

(20) $$\rho_{k}^{\mathrm{agg}}=\underset{i}{\mathrm{median}}\rho_{k,i}.$$

The aggregate residual is mapped to a gate

(21) $$\gamma_{k}=\left\{\begin{array}{cc} 0, & \rho_{k}^{\mathrm{agg}}\leq \tau_{0},\\ 1, & \rho_{k}^{\mathrm{agg}}\geq \tau_{1},\\ \frac{\rho_{k}^{\mathrm{agg}}-\tau_{0}}{\tau_{1}-\tau_{0}}, & \mathrm{otherwise}, \end{array}\right.$$

where $\tau_{0}$ and $\tau_{1}$ are lower and upper residual thresholds. A value $\gamma_{k}=0$ indicates a normal update, whereas $\gamma_{k}=1$ indicates strong LoS/NLoS interference. The weights are blended as

(22) $$w_{k}^{\mathrm{blend}}=\left(1-\gamma_{k}\right)w_{\mathrm{uni}}+\gamma_{k}w_{k}^{\mathrm{QP}},$$

where $w_{k}^{\mathrm{QP}}$ is the current reliability vector obtained from Algorithm 1. A per-anchor hold timer then suppresses persistently unreliable propagations:

(23) $$\begin{array}{cc} h_{k,i} & =\left\{\begin{array}{cc} H_{h}, & \rho_{k,i}>\tau_{R},\\ \max(h_{k-1,i}-1,0), & \mathrm{otherwise}, \end{array}\right.\\ {\tilde{w}}_{k,i} & =I\left(h_{k,i}=0\right)w_{k,i}^{\mathrm{blend}},\;w_{k,i}=\frac{{\tilde{w}}_{k,i}}{\sum_{j}{\tilde{w}}_{k,j}}. \end{array}$$

Here, $h_{k,i}$ is the hold counter of anchor *i*, $H_{h}$ is the hold duration measured in samples, $\tau_{R}$ is the per-anchor residual threshold, and I(·) is the indicator function. If all propagations are temporarily suppressed, the implementation falls back to $w_{\mathrm{uni}}$ to avoid division by zero.
**Algorithm 1** Projected gradient descent for propagation reliability weights.**Require:** Window data ${\left\{\delta_{t}^{\mathrm{imu}},\ell_{t}\right\}}_{t\in W_{k}}$, parameters $\lambda_{w},\beta$, step size η, maximum iterations $N_{\mathrm{pgd}}$, tolerance ϵ.**Ensure:** Current propagation reliability weights $w_{k}^{\mathrm{QP}}$.
  1:Initialize all $w_{t}$ from the previous solution or $w_{\mathrm{uni}}$.  2:**for** j=1 to $N_{\mathrm{pgd}}$ **do**  3:    Compute $\nabla_{w_{t}}L$ using Equation (16).  4:    $q_{t}\leftarrow w_{t}-\eta \nabla_{w_{t}}L$.  5:    $w_{t}\leftarrow \Pi_{\Delta }\left(q_{t}\right)$ using Equation (17).  6:    **if** relative objective decrease <ϵ **then**  7:        **break**  8:    **end if**  9:**end for**10:**return** $w_{k}^{\mathrm{QP}}$.

The bias-compensated UWB track coordinate is then obtained from

(24) $$\begin{array}{cc} \left\{{\bar{x}}_{k},{\hat{b}}_{k}\right\}=\underset{x\in I_{k},\;b\in {\left[0,b_{\max}\right]}^{M}}{\mathrm{arg min}}\;\; & \sum_{i=1}^{M}w_{k,i}{\left(r_{i}\left(x\right)+b_{i}-d_{k,i}\right)}^{2}+\lambda_{p}{\left(x-x_{k}^{\mathrm{pred}}\right)}^{2}\\ & +\lambda_{b}\sum_{i=1}^{M}{\left(b_{i}-{\hat{b}}_{k-1,i}\right)}^{2}, \end{array}$$

where ${\bar{x}}_{k}$ is the preliminary UWB-fused track coordinate, ${\hat{b}}_{k}={\left[{\hat{b}}_{k,1},\ldots ,{\hat{b}}_{k,M}\right]}^{⊤}$ is the estimated excess-range bias vector, $b_{\max}$ bounds physically plausible NLoS excess range, $\lambda_{b}$ imposes a first-order Markov smoothness prior on the bias state, and $I_{k}=\left[\max\left(x_{\min},x_{k}^{\mathrm{pred}}-W_{x}\right),\min\left(x_{\max},x_{k}^{\mathrm{pred}}+W_{x}\right)\right]$ is the local search interval. For a fixed *x*, each bias has the closed-form bounded update

(25) $$b_{i}^{★}\left(x\right)=\Pi_{\left[0,b_{\max}\right]}\left(\frac{w_{k,i}\left(d_{k,i}-r_{i}\left(x\right)\right)+\lambda_{b}{\hat{b}}_{k-1,i}}{w_{k,i}+\lambda_{b}}\right).$$

Substituting Equation (25) into Equation (24) leaves a one-dimensional bounded search over *x*. The closed-form bias update preserves a deterministic one-dimensional online search.

Finally, ${\bar{x}}_{k}$ is used as a scalar pseudo-measurement to correct the expanded state. Its variance is approximated from the local track-domain information as

(26) $$R_{k}^{x}=\mathrm{clip}\left[{\left(\lambda_{p}+\sum_{i=1}^{M}w_{k,i}\frac{j_{i}^{2}\left({\bar{x}}_{k}\right)}{\sigma_{\mathrm{LoS}}^{2}}\right)}^{-1},R_{\min}^{x},R_{\max}^{x}\right],\;j_{i}\left(x\right)=\frac{x-x_{i}}{r_{i}\left(x\right)},$$

where $R_{\min}^{x}$ and $R_{\max}^{x}$ are numerical lower and upper bounds on the pseudo-measurement variance. The Kalman correction is

(27) $$\begin{array}{cc} K_{k} & =P_{k|k-1}H^{⊤}{\left(HP_{k|k-1}H^{⊤}+R_{k}^{x}\right)}^{-1},\\ X_{k|k} & =X_{k|k-1}+K_{k}\left({\bar{x}}_{k}-HX_{k|k-1}\right),\\ P_{k|k} & =\left(I_{2}-K_{k}H\right)P_{k|k-1}{\left(I_{2}-K_{k}H\right)}^{⊤}+K_{k}R_{k}^{x}K_{k}^{⊤}, \end{array}$$

where $I_{2}$ is the 2×2 identity matrix. Because the second component of $K_{k}$ is generally nonzero after propagation, the UWB-fused position update corrects the velocity estimate as well as the position estimate. The final output is ${\hat{p}}_{k}={\left[HX_{k|k},y_{0}\right]}^{⊤}$.

Algorithm 2 summarizes the complete online estimator and shows how the prediction, reliability estimation, NLoS-control mechanisms, and closed-loop position–velocity correction are executed at each update.
**Algorithm 2** Online track-constrained UWB/IMU localization.**Require:** Anchors ${\left\{s_{i}\right\}}_{i=1}^{M}$, centerline T, UWB ranges $\left\{d_{k,i}\right\}$, IMU acceleration $\left\{u_{k}\right\}$, implementation parameters, update stride *S*.**Ensure:** Estimated trajectory $\left\{{\hat{p}}_{k}\right\}$.
  1:Initialize $X_{0|0}\leftarrow {\left[x_{\min},0\right]}^{⊤}$, $P_{0|0}$, ${\hat{b}}_{0}\leftarrow 0$, $w^{\mathrm{QP}}\leftarrow w_{\mathrm{uni}}$, and $h_{0,i}\leftarrow 0$.  2:**for** k=1 to *K* **do**  3:    Predict $X_{k|k-1}$, $P_{k|k-1}$, and $x_{k}^{\mathrm{pred}}$ by Equation (12).  4:    Recover $x_{k,i}^{\mathrm{inv}}$ by Equation (13) and compute increments by Equation (14).  5:    **if** mod(k,S)=0 **then**  6:        Solve Equation (15) with Algorithm 1 and obtain $w_{k}^{\mathrm{QP}}$.  7:    **end if**  8:    Compute $\rho_{k,i}$, $\rho_{k}^{\mathrm{agg}}$, and $\gamma_{k}$ by Equations (19)–(21).  9:    Blend, hold suppress, and renormalize weights by Equations (22) and (23).10:    Solve the bias-augmented track update in Equation (24) and obtain ${\bar{x}}_{k},{\hat{b}}_{k}$.11:    Compute $R_{k}^{x}$ by Equation (26); correct $X_{k|k}$ and $P_{k|k}$ by Equation (27).12:    Output ${\hat{p}}_{k}={\left[HX_{k|k},y_{0}\right]}^{⊤}$.13:**end for**

## 5. Design Rationale and Complexity

### 5.1. Difference from Conventional Fusion and Robust Filtering

A conventional EKF with direct UWB ranges can be written as

(28) $${\hat{x}}_{k}^{+}={\hat{x}}_{k}^{-}+K_{k}\left(d_{k}-h\left({\hat{x}}_{k}^{-}\right)\right).$$

If an anchor has a positive NLoS excess range, the expected correction contains a term proportional to $K_{k}b_{k}$. Under narrow-path anchor deployment, this residual is easily mapped into poorly observed geometric directions or into a biased longitudinal correction. Adaptive covariance inflation can reduce the gain, but it also suppresses useful LoS information and does not identify which anchor–target propagation is unreliable.

The proposed scheme separates propagation reliability evaluation from state closure. The IMU provides the short-window motion reference in Equation (15), and motion consistency determines the propagation weights. Residual gating, hold suppression, and excess-range bias compensation then produce the scalar pseudo-measurement ${\bar{x}}_{k}$ through Equation (24). The Kalman correction in Equation (27) uses this screened pseudo-measurement to update $X_{k}={\left[x_{k},v_{k}\right]}^{⊤}$, thereby closing the velocity loop and limiting open-loop inertial drift.

The proposed reliability estimation also differs from robust least-squares and trimmed estimators. Robust losses and trimming rules usually suppress measurements according to snapshot residual magnitude and are effective when corrupted propagations are sparse and clearly separable. When NLoS bias persists or affects multiple propagations in a correlated manner, several biased propagations can jointly support an incorrect longitudinal solution, so snapshot residuals alone may not identify the unreliable subset. The proposed method introduces an independent temporal reference from the IMU-predicted motion and evaluates the motion implied by each UWB propagation before the final update. This distinction is also examined experimentally in Section 7.2.

### 5.2. Component Roles

Table 2 summarizes the role of each module. The modules form a sequential estimation structure: the track centerline defines the one-dimensional state, per-anchor inversion produces UWB-derived motion increments, the IMU prior stabilizes the update, and the reliability and NLoS-control stages regulate the propagation contributions to track-domain fusion.

### 5.3. Parameter Roles

The following implementation parameters control the main stages of the proposed method. The search half-width $W_{x}$ controls recovery from short-term prediction error; $N_{x}$ controls the discretization of the one-dimensional solve. The prior weight $\lambda_{p}$ balances IMU stabilization and UWB correction, while $\lambda_{b}$ and $b_{\max}$ control the temporal smoothness and physical range of the propagation-specific excess-range bias. The process-noise parameters $q_{x}$ and $q_{v}$ determine how quickly the closed-loop state can adapt between UWB updates, and $R_{\min}^{x},R_{\max}^{x}$ bound the pseudo-measurement variance used in Equation (27). The window length $N_{w}$ trades reliability-estimation variance against reaction delay. The smoothness weight $\lambda_{w}$ reduces oscillation, and β prevents over-concentrated weights. The thresholds $\tau_{0}$, $\tau_{1}$, and $\tau_{R}$ are set according to the gap between LoS residual statistics and residuals caused by NLoS propagation.

### 5.4. Practical Parameter Calibration Procedure

For deployment in a new rail-guided environment, a short calibration procedure is used to select the main parameters of the proposed method. The procedure uses three types of prior information: the anchor geometry and track map, a short LoS ranging sequence, and the expected motion and blockage characteristics of the vehicle.

First, the UWB LoS noise level is estimated from a short calibration run or a static measurement period. Let ${\hat{\sigma }}_{\mathrm{LoS}}$ denote the standard deviation of the LoS ranging residuals, and let $q_{0.95}$ and $q_{0.99}$ denote their empirical 95th and 99th percentiles. The residual thresholds can then be initialized as $\tau_{0}\approx q_{0.95}$ and $\tau_{1}\approx q_{0.99}$, while the per-propagation residual threshold $\tau_{R}$ is set between the upper LoS residual range and the expected NLoS excess range. In practice, $\tau_{R}$ should be large enough to avoid suppressing normal LoS fluctuations but small enough to react to persistent positive range errors.

Second, the search half-width $W_{x}$ is selected according to the maximum possible short-term prediction error. Given the maximum speed $v_{\max}$, acceleration uncertainty, sampling period Δt, and the update stride *S*, $W_{x}$ should cover the largest expected drift of the IMU prediction between UWB corrections, with an additional margin for UWB noise. A larger $W_{x}$ improves recovery from temporary prediction errors but increases the risk of branch switching in geometrically ambiguous regions.

Third, the process-noise parameters $q_{x}$ and $q_{v}$ are determined by the IMU quality and the expected vehicle dynamics. A higher-grade IMU or smoother rail-guided motion allows smaller process noise, whereas low-cost IMU measurements or stronger vibration require larger $q_{x}$ and $q_{v}$. The prior strength $\lambda_{p}$ is then chosen to keep the track-domain update close to the IMU prediction when only a few reliable UWB propagations remain, without preventing correction by consistent UWB observations.

Fourth, the window length $N_{w}$, weight-update stride *S*, and hold duration $H_{h}$ are selected according to the expected NLoS time scale and the available computation budget. The window should be long enough to capture a stable motion-consistency trend but short enough to respond to a change in propagation state. The interval SΔt between two weight updates should remain shorter than the expected propagation-state transition time. Short intermittent blockage favors smaller $N_{w}$ and $H_{h}$, whereas persistent tunnel, platform, or vehicle blockage permits larger values for more stable suppression.

Fifth, the bias parameters $b_{\max}$ and $\lambda_{b}$ are selected according to the expected NLoS excess range. The upper bound $b_{\max}$ should cover the largest physically plausible positive range bias in the environment, while $\lambda_{b}$ controls how quickly the estimated excess range is allowed to vary. A larger $\lambda_{b}$ is suitable for slowly varying NLoS bias, whereas a smaller value allows faster adaptation.

The pseudo-measurement variance bounds $R_{\min}^{x}$ and $R_{\max}^{x}$ are initialized from the empirical distribution of track-domain position uncertainty during the LoS calibration run. The lower bound prevents unrealistically high confidence in favorable geometry, whereas the upper bound prevents a single uncertain pseudo-measurement from destabilizing the state update. Finally, the numerical parameters $N_{x}$, η, and $N_{\mathrm{pgd}}$ are implementation parameters. The grid number $N_{x}$ is selected according to the required longitudinal resolution within the search interval. The PGD step size η and maximum iteration number $N_{\mathrm{pgd}}$ are chosen to ensure stable decrease in the convex objective in Equation (15) under the deployed number of anchors. After convergence and real-time feasibility are verified, these numerical parameters remain fixed across propagation environments.

### 5.5. Online Complexity

The per-step prediction, inversion, residual computation, and closed-loop covariance correction have complexity O(M) or O(1). The bias-augmented one-dimensional update has complexity $O(N_{x}M)$ for a grid search with $N_{x}$ points because the bias variables have the closed-form update in Equation (25) for each candidate *x*. The PGD reliability update costs $O(N_{\mathrm{pgd}}N_{w}M)$ when executed and $O(N_{\mathrm{pgd}}N_{w}M/S)$ amortized over update stride *S*. Thus the proposed per-step complexity is

(29) $$O\left(N_{x}M\right)+O\left(N_{\mathrm{pgd}}N_{w}M/S\right),$$

which is deterministic for fixed parameters.

Table 3 summarizes the practical selection rules for the main parameter groups.

## 6. Simulation Setup and Baselines

### 6.1. Simulation Parameters

The simulation follows a rail transportation scenario where the mobile target moves along a known track centerline. All simulations, data processing, and runtime measurements were performed using Python 3.11 on a computer equipped with a 13th Gen Intel Core i5-13500H processor (12 cores and 16 threads; Intel Corporation, Santa Clara, CA, USA), 16 GB of RAM, and a 64-bit Windows 11 Pro operating system (Microsoft Corporation, Redmond, WA, USA). Table 4 summarizes the main parameters. The track interval is x∈[0,300] m with $y_{0}=2.5$ m, and four UWB anchors are deployed at (0,0), (100,5), (200,0), and (300,5) m. The sampling period is Δt=0.02 s, corresponding to 50 Hz operation. The LoS ranging noise standard deviation is $\sigma_{\mathrm{LoS}}=0.02$ m. In dynamic localization, the target first accelerates at $0.5\;m/s^{2}$ and then decelerates at $-0.5\;m/s^{2}$ after 24 s. Unless otherwise stated, NLoS propagation occurs during 40–45 s with (μ,σ)=(0.5,0.1) m.

Table 5 lists the main hyperparameters of the proposed method. Failure thresholds are $e_{\mathrm{fail}}=5$ m for static localization and $e_{\mathrm{fail}}=0.5$ m for dynamic localization.

### 6.2. Fair Track-Domain Baselines

To ensure fair comparison, all the methods in the experiments are implemented in the same one-dimensional track domain. In other words, every method estimates only the longitudinal coordinate *x* on the known track centerline and outputs $\hat{p}={\left[\hat{x},y_{0}\right]}^{⊤}$. Thus, the reported RMSE reflects algorithmic robustness in the track domain rather than the advantage of constraining only the proposed method.

For static localization, we compare the proposed static estimator with two track-domain baselines. Track-1D-LS solves the one-dimensional least-squares ranging problem on the track. Track-Robust LS uses a trimmed robust least-squares objective on the track to reduce the influence of one unreliable propagation. For dynamic localization, we compare with Track-EKF, Track-Gauss-AUKF, Track-Adaptive KF, and Track-SW-FGO. These methods follow the corresponding filtering or smoothing ideas, but their states and observations are expressed in the one-dimensional track coordinate. All the methods use the same UWB ranging results, IMU measurements, anchor deployment, sampling rate, and Monte Carlo trials. For compact figure legends, Track-EKF, Track-Gauss-AUKF, Track-Adaptive KF, and Track-SW-FGO are denoted by T-EKF, T-Gauss, T-AKF, and T-FGO, respectively.

To characterize the gain provided by propagation reliability estimation and the associated stabilization mechanisms, we additionally implement four simpler track-domain alternatives: robust 1D UWB filter, adaptive covariance KF, trimmed LS + IMU smoothing, and bias-aware EKF. These alternatives represent robust measurement weighting, global covariance adaptation, residual trimming with inertial smoothing, and bias-aware filtering without explicit propagation reliability estimation. They are evaluated under both the default single-link NLoS setting and the Correlated-2link NLoS setting.

### 6.3. Evaluation Criteria

Static experiments evaluate the default mixed LoS/NLoS scenario and sweeps of NLoS ranging-error parameters. Dynamic experiments evaluate LoS scenarios, mixed LoS/NLoS scenarios, two IMU noise levels, additional LoS/NLoS interference models, hyperparameter sensitivity, and component analysis. We report RMSE, median, P95, maximum error, and failure rate when informative. When all failure rates are zero, the analysis focuses on RMSE, P95, and maximum error to avoid reporting non-informative metrics.

## 7. Experimental Results

We first evaluate mixed LoS/NLoS scenarios because they directly test NLoS mitigation under fair track-domain baselines. We then compare the proposed method with simpler alternatives to quantify the gain from propagation reliability estimation and the associated stabilization mechanisms. After that, we report LoS scenarios, IMU-noise sensitivity, additional LoS/NLoS interference models, hyperparameter sensitivity, runtime, component analysis, a real-data feasibility test, and static verification. For readability, the time-series curves are lightly downsampled in the figures, while all the tabulated metrics are computed from the original samples.

### 7.1. Dynamic Localization in Mixed LoS/NLoS Scenarios

**Figure 4:**
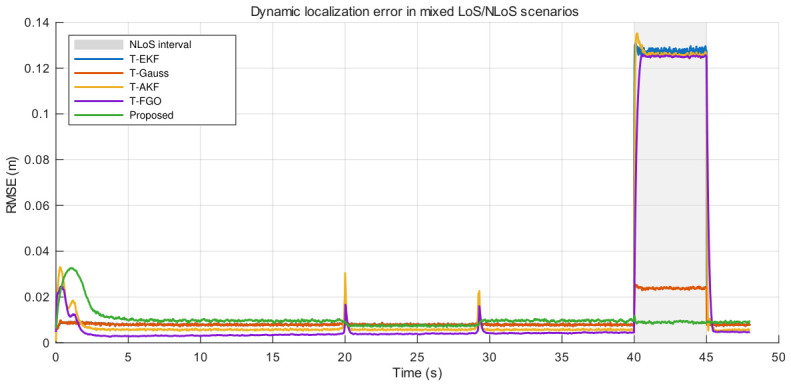
Dynamic localization error in mixed LoS/NLoS scenarios.

Figure 4 and Table 6 report the default mixed LoS/NLoS scenario. All the methods estimate the same one-dimensional track coordinate, and one anchor–target propagation is affected by NLoS propagation during 40–45 s. The proposed method achieves an overall RMSE of 0.0108 m and an NLoS-interval RMSE of 0.0090 m. Relative to Track-EKF, Track-Gauss-AUKF, Track-Adaptive KF, and Track-SW-FGO, the NLoS-interval RMSE is reduced by 92.9%, 62.1%, 92.8%, and 92.6%, respectively. The shaded region in Figure 4 shows that the baselines respond to the NLoS duration with clear error bursts, whereas the proposed method keeps the error close to its LoS level. Under the common track constraint, propagation reliability estimation reduces the error burst produced by the NLoS-affected propagation.

The degradation patterns are consistent with how the methods treat the biased range. Track-EKF directly maps the NLoS-contaminated innovation into the state correction, while Track-Adaptive KF reduces the gain of the overall UWB update and therefore weakens reliable LoS information together with the corrupted propagation. Track-SW-FGO smooths the trajectory over a temporal window, but a persistent biased factor remains active throughout that window. Track-Gauss-AUKF performs better because its adaptive noise model limits large innovations, although it does not explicitly determine which propagation is unreliable. In the proposed method, reliability is evaluated before the absolute state update; the affected propagation is down-weighted, the slowly varying positive excess range is absorbed by the bias state, and hold suppression prevents premature re-entry. This explains why the NLoS error remains close to the LoS level. The reported 0.0090 m value represents longitudinal accuracy under the stated simulated noise and ideal centerline; practical two-dimensional accuracy additionally depends on map error and lateral sway, as quantified in Section 7.6.

### 7.2. Comparison with Simpler Alternatives

We compare the proposed method with four simpler alternatives: robust 1D UWB filter, adaptive covariance KF, trimmed LS + IMU smoothing, and bias-aware EKF. All the methods are implemented in the same one-dimensional track domain and use the same UWB ranges, IMU measurements, anchor deployment, sampling rate, and evaluation protocol.

**Figure 5:**
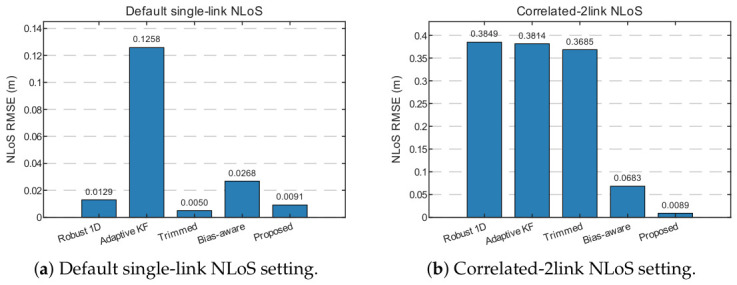
NLoS-interval RMSE comparison with simpler alternatives.

Table 7 and Figure 5 summarize the results under the default single-link NLoS setting and the Correlated-2link NLoS setting. In the default single-link NLoS case, trimmed LS + IMU smoothing achieves the lowest NLoS-interval RMSE of 0.0050 m, compared with 0.0091 m for the proposed method. Single-residual trimming is effective in this setting because removing the corrupted observation leaves sufficient reliable longitudinal information.

The performance separation becomes pronounced in the Correlated-2link NLoS setting. Robust 1D UWB filter, adaptive covariance KF, and trimmed LS + IMU smoothing reach NLoS-interval RMSE values of 0.3849 m, 0.3814 m, and 0.3685 m, respectively. Bias-aware EKF reduces the NLoS-interval RMSE to 0.0683 m, while the proposed method achieves 0.0089 m. In the Correlated-2link setting, one biased propagation remains after single-residual trimming, and the corrupted propagations can support a mutually consistent but incorrect snapshot solution. The IMU-based temporal reference supplies an additional cue beyond snapshot residual magnitude. The resulting gain is concentrated when NLoS bias persists or affects multiple propagations in a correlated manner, while residual trimming remains effective for isolated single-link interference.

### 7.3. Dynamic Localization in LoS Scenarios

**Figure 6:**
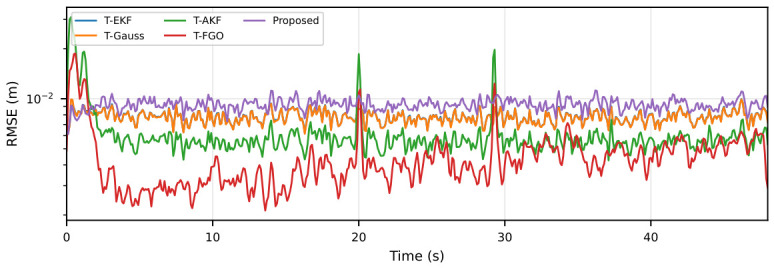
Dynamic localization error in LoS scenarios.

Figure 6 and Table 8 show dynamic localization in LoS scenarios. All the methods achieve centimeter-level accuracy under the common track constraint. Track-SW-FGO gives the lowest average RMSE of 0.005 m by smoothing a window of consistently reliable measurements. The proposed method gives an RMSE of 0.009 m and a maximum error of 0.033 m; its reliability regularization, bias state, and gating logic add little information when every propagation is reliable and introduce a small response delay. These results position the proposed framework as a robustness-oriented estimator for LoS/NLoS transitions and NLoS intervals.

### 7.4. Sensitivity to IMU Noise

**Figure 7:**
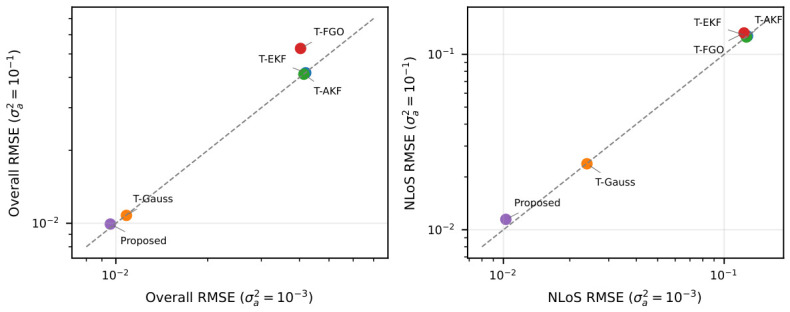
Localization performance under different IMU noise levels.

Figure 7 and Table 9 compare two IMU acceleration-noise variances, $10^{-3}$ and $10^{-1}\;m^{2}/s^{4}$. The proposed method gives NLoS-interval RMSE values of 0.0103 m and 0.0115 m, respectively. Within the tested IMU-noise range, the small change follows from the use of short-term IMU motion increments for propagation reliability estimation and the repeated velocity correction in Equation (27). Two mechanisms limit the accumulation of inertial error. First, the reliability estimator uses short-window displacement increments rather than an unconstrained long-term inertial trajectory. Second, the position–velocity state is repeatedly corrected by the UWB-derived pseudo-measurement. The IMU therefore supplies the local motion trend used for comparison, while absolute position remains constrained by UWB. Reliable propagation ordering requires the short-term IMU bias to remain smaller than the differences among the UWB-derived increments.

The overall RMSE values in Table 9 follow the same trend: the proposed method gives the smallest overall RMSE at both IMU noise levels and keeps the maximum error below 0.11 m.

### 7.5. Additional LoS/NLoS Interference Models

**Figure 8:**
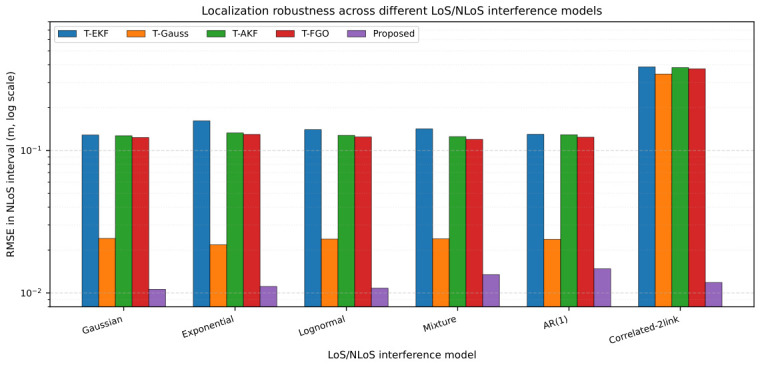
RMSE in the NLoS interval under additional LoS/NLoS interference models. A logarithmic vertical axis is used to show both centimeter-level and decimeter-level errors without dense numerical labels.

To evaluate distributional and temporal robustness, we consider six LoS/NLoS interference models: Gaussian, exponential, lognormal, Gaussian mixture, temporally correlated AR(1), and Correlated-2link blockage. Table 10 and Figure 8 report NLoS-interval RMSE. The NLoS-interval RMSE of the proposed method remains between 0.0106 m and 0.0148 m across all six cases. In the first five models, Track-Gauss-AUKF is the closest baseline, with RMSE values around 0.022–0.024 m. In the Correlated-2link case, all the baselines degrade substantially; the closest baseline reaches 0.3438 m, while the proposed method remains at 0.0118 m. The six-model comparison demonstrates robustness across distribution shape, temporal correlation, and simultaneous blockage of multiple propagations.

The similar results under Gaussian, exponential, lognormal, and mixture models show that motion inconsistency, rather than a predefined NLoS probability density, drives the reliability measure. The AR(1) case is more difficult because a slowly varying bias can produce smooth increments that partially resemble valid target motion, which explains the modest increase in the proposed RMSE to 0.0148 m and highlights the role of the explicit bias state. The Correlated-2link case is fundamentally different: multiple biased propagations change together and dominate the available geometry, so global covariance adaptation or temporal smoothing cannot recover a reliable subset. The large separation between the proposed method and the baselines in this case is consistent with the value of propagation-specific weighting before the state update.

### 7.6. Hyperparameter Sensitivity, Centerline Error, and Runtime

**Figure 9:**
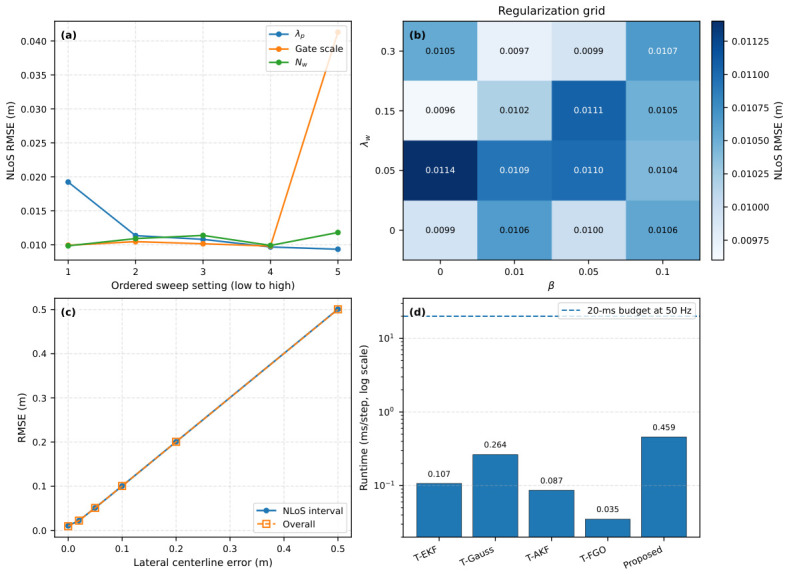
Hyperparameter sensitivity, centerline error, and runtime. (**a**) NLoS RMSE across ordered sweep settings; the tested values are listed in Table 11. (**b**) NLoS RMSE over the $\lambda_{w}$–β grid, shown to four decimal places. (**c**) Overall and NLoS-interval RMSE versus lateral centerline error; the near overlap of the curves indicates direct error transfer. (**d**) Mean runtime per update on a logarithmic scale; the dashed line is the 20 ms budget corresponding to 50 Hz sampling.

Table 11 and Figure 9 summarize the hyperparameter sensitivity, track-centerline sensitivity, and runtime tests. The sensitivity results complement the calibration procedure in Section 5.4: the calibration procedure provides initial parameter values from LoS residual statistics, motion constraints, and expected blockage duration, while the sensitivity test verifies that the estimator remains stable around these practical settings. The window length $N_{w}$ and regularization parameters $\lambda_{w},\beta$ mainly control the temporal smoothness of the reliability sequence, so moderate changes alter estimation variance more than final propagation selection. In contrast, the residual-gating scale directly determines when the estimator switches from uniform UWB fusion to reliability-weighted NLoS mitigation. A loose gate admits contaminated ranges, whereas an overly strict gate may suppress valid LoS information; this explains the larger 0.010–0.041 m RMSE range. The prior strength $\lambda_{p}$ balances inertial continuity and UWB correction: too little prior permits abrupt branch changes, while too much prior delays correction of inertial prediction error. The centerline-offset test in Table 12 shows that a hard track constraint requires a reliable track map: an offset in the track centerline is transferred almost directly into localization error. Table 13 and Figure 9d show a mean proposed-method runtime of 0.459 ms per update. The ratio 20/0.459=43.6 means that the computation uses approximately 2.3% of the 20 ms time budget associated with 50 Hz sampling.

### 7.7. Component Analysis Under Multiple NLoS-Affected Propagations

**Figure 10:**
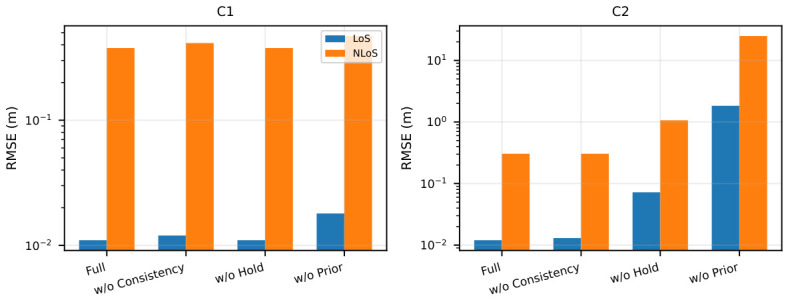
Component analysis under multiple NLoS-affected propagations.

We evaluate the role of each component when multiple anchor–target propagations are affected by NLoS during 40–45 s. Scenario C1 contains two NLoS-affected propagations with μ=1.0 m, while C2 contains three NLoS-affected propagations with μ=2.5 m. Table 14 and Figure 10 compare the full method with variants without consistency learning, hold suppression, or the IMU-centered prior.

The dominant stabilization mechanism changes with the number of corrupted propagations. In C1, where two propagations are affected by NLoS, removing consistency learning increases the NLoS-interval RMSE from 0.377 m to 0.413 m, showing that motion consistency provides useful discrimination while a valid LoS subset remains. In C2, three of four propagations suffer from severe NLoS, and the full method (0.305 m) approaches the variant without consistency learning because the corrupted propagations dominate the increment set. The IMU-centered prior and hold suppression then provide the main stabilization. Removing the IMU prior increases the NLoS-interval RMSE to 24.863 m, while removing hold suppression raises it to 1.065 m.

### 7.8. Real-Data Feasibility Test on Selected STAR-Loc Straight Segments

We conduct a real-data stress test using the public STAR-loc dataset [27]. STAR-loc provides general-purpose indoor UWB/IMU sequences with asynchronous measurements, lateral motion, and practical sensor noise. We fit a local three-dimensional centerline to selected approximately straight trajectories and project the measurements onto a one-dimensional track-like coordinate to evaluate the estimator under these conditions.

The dataset-provided calibrated UWB ranges are synchronized to a 20 Hz grid by nearest-neighbor sampling for each anchor. Crucially, the IMU acceleration is rotated by the provided quaternion, projected onto the fitted centerline, and used directly in a strict online protocol; no future IMU samples, subsegment-wise mean removal, or non-causal smoothing are used.

Table 15 lists the selected real-data subsegments. Notably, these segments exhibit natural lateral sway (RMS errors of 0.025–0.111 m) and high measurement noise (calibrated UWB range RMSE of 0.122–0.266 m), presenting a significantly harsher condition than the ideal simulated track.

**Figure 11:**
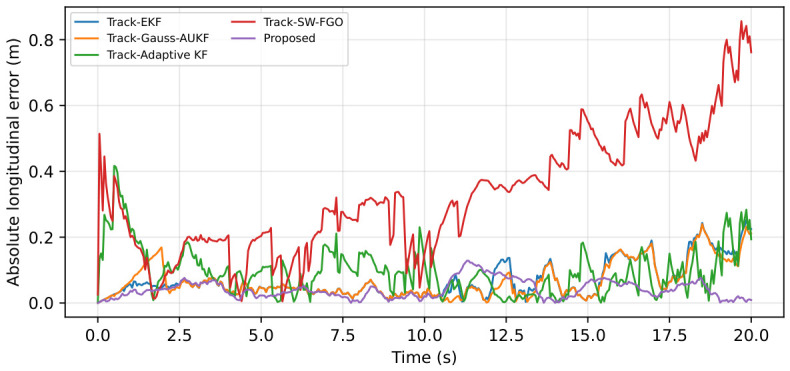
Representative STAR-loc straight-segment test on S2. The curves show absolute longitudinal error after projecting the real UWB/IMU data to the fitted local centerline.

Table 16 and Figure 11 summarize the localization results. Under this strict online calibrated-range protocol, the proposed method successfully maintains track-domain stability, achieving an average longitudinal RMSE of 0.0555 m. In contrast, the baseline methods suffer severe degradation, yielding RMSEs of 0.1266 m for Track-EKF, 0.8290 m for Track-Gauss-AUKF, 0.1610 m for Track-Adaptive KF, and 0.4099 m for Track-SW-FGO. The corresponding RMSE reductions are 56.1%, 93.3%, 65.5%, and 86.4%, respectively. These results provide strong empirical evidence that propagation reliability estimation and closed-loop position–velocity correction remain effective for real asynchronous UWB/IMU data streams.

Track-Gauss-AUKF reaches an RMSE of 0.8290 m in the real-data test despite its strong performance in the simulated Gaussian-error settings. This behavior is related to the innovation-covariance matching mechanism of adaptive UKF-type algorithms. In the STAR-loc dataset, asynchronous measurements are affected by frequent, non-Gaussian, and abrupt multipath jumps. These real-world anomalies may cause the adaptive measurement covariance to inflate or fluctuate rapidly, leading to unstable sigma-point sampling and weakening the local Gaussian approximation. The proposed method evaluates reliability in the motion-increment domain and absorbs large positive range errors through explicit bias states, which improves resilience to unmodeled real-world measurement noise.

### 7.9. Static Verification Under NLoS Propagation

**Figure 12:**
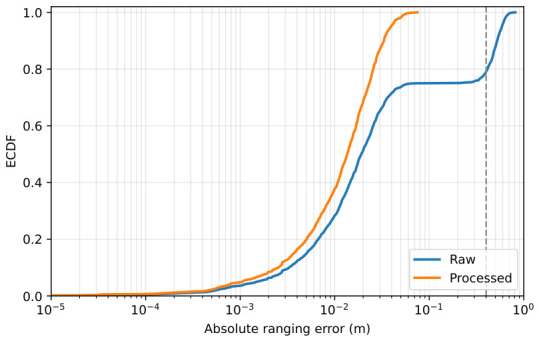
ECDF of absolute ranging error.

The static test quantifies propagation screening using an outlier rate defined as the fraction of absolute ranging errors above $\tau_{R}=0.4$ m. Figure 12 and Table 17 show an unscreened RMSE of 0.255 m and an outlier rate of 20.9%. After screening, the RMSE decreases to 0.020 m, no retained sample exceeds 0.4 m, and the remaining P95 of 0.039 m characterizes the residual ranging uncertainty.

Table 18 summarizes the single-snapshot static localization and parameter sweeps. Track-Robust LS achieves the lowest error because sparse corruption can be removed directly through the largest snapshot residual. The proposed estimator derives its additional information from temporal UWB/IMU consistency, which becomes informative in dynamic localization.

## 8. Conclusions and Future Work

This article proposed a track-constrained UWB/IMU fusion localization framework with motion-consistency-based propagation reliability estimation for rail transportation scenarios. First, the localization task was formulated as a one-dimensional track-constrained problem, converting UWB ranges into candidate longitudinal coordinates to mitigate geometric ill-conditioning. Second, the reliability of each anchor–target propagation was dynamically estimated over a sliding window by comparing UWB-derived motion increments with IMU-predicted short-term kinematics. Third, this reliability metric was integrated into a bias-augmented track-domain update—supported by residual gating and hold suppression—prior to a closed-loop position–velocity Kalman correction to effectively mitigate LoS/NLoS interference.

Comprehensive evaluations under a unified track constraint revealed the specific operational boundaries and advantages of the proposed framework. While simpler alternatives, such as trimmed smoothing or factor-graph optimization, are sufficient in benign line-of-sight (LoS) or isolated single-link NLoS conditions, the proposed multi-stage architecture demonstrates its necessity under persistent or correlated multi-link NLoS blockages. From an engineering perspective, this additional complexity is highly justified when continuous localization is required but dense balise infrastructure or distinctive LiDAR features are unavailable. In the default mixed LoS/NLoS scenario, the proposed method achieved an NLoS-interval RMSE of 0.0090 m over 500 Monte Carlo trials, yielding error reductions of 92.9%, 62.1%, 92.8%, and 92.6% relative to Track-EKF, Track-Gauss-AUKF, Track-Adaptive KF, and Track-SW-FGO, respectively. This robustness was maintained across six distinct NLoS interference models, with NLoS-interval RMSEs consistently bounded between 0.0106 m and 0.0148 m. Furthermore, runtime measurements confirmed online feasibility at 50 Hz, and the provided calibration procedure established a practical deployment route. Ultimately, a real-data stress test on the STAR-loc dataset yielded an average longitudinal RMSE of 0.0555 m, validating the estimator’s practical applicability against real-world lateral sway, unmodeled non-Gaussian noise, and asynchronous measurements.

The current real-data evaluation utilizes five approximately straight STAR-loc trajectory segments represented by fitted local centerlines. To achieve a comprehensive field validation, future work will focus on developing a customized rail-guided UWB/IMU platform. This will enable broader evaluations across longer operating distances, diverse anchor geometries, and controlled multi-link NLoS conditions. Methodologically, we plan to extend the track-domain formulation to support curved centerlines and incorporate probabilistic map constraints to systematically handle lateral motion and map uncertainties.

## Figures and Tables

**Table 1:** Principal abbreviations used in this article.

Abbreviation	Meaning	Abbreviation	Meaning
AGV	Automated guided vehicle	UWB	Ultra-wideband
IMU	Inertial measurement unit	GNSS	Global navigation satellite system
ToA	Time of arrival	RFID	Radio-frequency identification
SLAM	Simultaneous localization and mapping	ECDF	Empirical cumulative distribution function
LoS	Line-of-sight	NLoS	Non-line-of-sight
KF	Kalman filter	EKF	Extended Kalman filter
UKF	Unscented Kalman filter	AUKF	Adaptive unscented Kalman filter
FGO	Factor-graph optimization	PGD	Projected gradient descent
RMSE	Root-mean-square error	P95	95th-percentile error
MAE	Mean absolute error	QP	Quadratic program

**Table 2:** Functions and typical failure modes of the main estimator components.

Component	Primary Role	Typical Failure If Removed
Track centerline	Reduces the state dimension and removes the weak lateral mode	Two-dimensional estimates drift along poorly observed directions
Per-anchor inversion	Converts each ranging result into a candidate longitudinal coordinate	Per-propagation motion consistency cannot be computed
IMU prior in Equation (24)	Stabilizes the one-dimensional update and prevents branch switching	Large jumps when reliable propagations are few or temporarily suppressed
Bias state in Equation (24)	Absorbs slowly varying positive NLoS excess range	Constant-bias NLoS can pass an increment test and corrupt absolute position
Closed-loop state correction	Uses the UWB-fused pseudo-measurement to correct both $x_{k}$ and $v_{k}$	Velocity becomes a pure open-loop inertial integral and drifts over time
Consistency term in Equation (15)	Estimates propagation reliability from IMU/UWB motion agreement	Unreliable propagations retain excessive weight before gating or hold suppression
Smoothness term $\lambda_{w}$	Reduces weight chattering	Weights and estimates become jittery
Uniform regularizer β	Prevents single-propagation weight collapse	Incorrect-propagation lock-in under ambiguity
Residual gate	Reacts quickly to abrupt residual growth	Sliding-window weights respond too slowly to sudden ranging error
Hold suppression	Keeps persistently unreliable propagations out for a minimum duration	Unreliable propagations repeatedly re-enter and cause oscillatory errors

**Table 3:** Practical calibration guideline for the main parameters.

Parameter Group	Parameters	Main Determining Factors	Practical Selection Rule
Track search	$W_{x}$, $N_{x}$	Anchor geometry, speed range, IMU prediction error	Set $W_{x}$ to cover the maximum short-term prediction drift plus a UWB noise margin; choose $N_{x}$ according to the required longitudinal resolution.
IMU prior and process model	$\lambda_{p}$, $q_{x}$, $q_{v}$	IMU quality, vehicle acceleration, vibration level	Use larger process noise for low-cost IMU or stronger vibration; increase $\lambda_{p}$ when UWB geometry is weak or reliable propagations are temporarily few.
Bias compensation	$b_{\max}$, $\lambda_{b}$	Expected NLoS excess range and its temporal variation	Set $b_{\max}$ to the largest plausible positive NLoS bias; increase $\lambda_{b}$ for slowly varying NLoS bias and decrease it for rapidly changing blockage.
Reliability window	$N_{w}$, *S*, $\lambda_{w}$, β	NLoS duration, sampling rate, geometric ambiguity	Choose $N_{w}$ to cover a stable motion-consistency trend; use $\lambda_{w}$ to suppress weight chattering and β to avoid single-propagation collapse.
Residual gating and hold	$\tau_{0}$, $\tau_{1}$, $\tau_{R}$, $H_{h}$	LoS residual statistics, NLoS duration, UWB noise level	Estimate $\tau_{0}$ and $\tau_{1}$ from LoS residual percentiles; set $\tau_{R}$ above normal LoS fluctuations; choose $H_{h}$ according to the expected blockage duration.
Pseudo-measurement variance	$R_{\min}^{x}$, $R_{\max}^{x}$	LoS track-domain uncertainty, local geometry	Use empirical LoS uncertainty to prevent overconfidence and cap the influence of uncertain pseudo-measurements.
Numerical solver	η, $N_{\mathrm{pgd}}$	Number of anchors, real-time budget	Use a step size that gives stable objective decrease and an iteration budget that satisfies the sampling-period constraint.

**Table 4:** Simulation setup.

Item	Value
Track interval	x∈[0,300] m, $y_{0}=2.5$ m
Anchors (M=4)	(0,0), (100,5), (200,0), (300,5) m
Sampling period	Δt=0.02 s (50 Hz)
LoS ranging noise	$\sigma_{\mathrm{LoS}}=0.02$ m
Static sweeps	μ∈[0,1] m, σ∈[0.1,0.5] m
Dynamic motion	$+0.5\;m/s^{2}$ then $-0.5\;m/s^{2}$ after 24 s
NLoS duration	40–45 s, one anchor, (μ,σ)=(0.5,0.1) m
IMU noise levels	$\sigma_{a}^{2}\in \left\{10^{-3},10^{-1}\right\}\;m^{2}/s^{4}$
Monte Carlo trials	Static: 500; all dynamic tests: 500 per setting

**Table 5:** Hyperparameters of the proposed method.

Parameter	Value
Search window half-width	$W_{x}=2$ m
Grid points per solve	$N_{x}=200$
Prior strength	$\lambda_{p}=0.5$
Bias smoothness	$\lambda_{b}=0.30$
Bias upper bound	$b_{\max}=3.0$ m
State process covariance	$q_{x}=2\times 10^{-4},\;q_{v}=10^{-2}$
Pseudo-measurement variance bounds	$R_{\min}^{x}=10^{-4},\;R_{\max}^{x}=4.0$
QP window length	$N_{w}=25$
Weight-update stride	S=5 steps
QP smoothness	$\lambda_{w}=0.15$
QP uniform regularizer	β=0.05
Gating thresholds	$\tau_{0}=0.25$ m, $\tau_{1}=1.0$ m
Residual threshold	$\tau_{R}=0.4$ m
PGD step size	η=0.15
PGD maximum iterations	$N_{\mathrm{pgd}}=60$
Hold duration	$H_{h}=200$ samples, corresponding to 4 s at Δt=0.02 s

**Table 6:** Dynamic localization summary in mixed LoS/NLoS scenarios.

Method	RMSE (m)	P95 (m)	Max (m)	${\mathrm{RMSE}}_{40–45}$ (m)
Track-EKF	0.042	0.127	0.219	0.127
Track-Gauss-AUKF	0.011	0.024	0.056	0.024
Track-Adaptive KF	0.041	0.126	0.191	0.126
Track-SW-FGO	0.040	0.124	0.157	0.123
Proposed	0.011	0.021	0.091	0.009

**Table 7:** Comparison with simpler alternatives. All errors are in meters.

**Default Single-Link NLoS**				
Method	RMSE (m)	P95 (m)	${\mathrm{RMSE}}_{40–45}$ (m)	${\mathrm{P95}}_{40–45}$ (m)
Robust 1D UWB filter	0.0087	0.0173	0.0129	0.0242
Adaptive covariance KF	0.0413	0.1260	0.1258	0.1485
Trimmed LS + IMU smoothing	0.0050	0.0097	0.0050	0.0097
Bias-aware EKF	0.0517	0.0321	0.0268	0.0518
Proposed	0.0109	0.0205	0.0091	0.0178
**Correlated-2link NLoS**				
Method	RMSE (m)	P95 (m)	${\mathrm{RMSE}}_{40–45}$ (m)	${\mathrm{P95}}_{40–45}$ (m)
Robust 1D UWB filter	0.1256	0.3827	0.3849	0.4695
Adaptive covariance KF	0.1240	0.3847	0.3814	0.4313
Trimmed LS + IMU smoothing	0.1198	0.3688	0.3685	0.4483
Bias-aware EKF	0.0575	0.0618	0.0683	0.1237
Proposed	0.0108	0.0205	0.0089	0.0175

**Table 8:** Dynamic localization summary in LoS scenarios.

Method	RMSE (m)	P95 (m)	Max (m)	${\mathrm{RMSE}}_{40–45}$ (m)
Track-EKF	0.008	0.016	0.033	0.008
Track-Gauss-AUKF	0.008	0.016	0.033	0.008
Track-Adaptive KF	0.007	0.013	0.061	0.006
Track-SW-FGO	0.005	0.009	0.044	0.005
Proposed	0.009	0.018	0.033	0.009

**Table 9:** Dynamic localization summary under two IMU noise levels.

IMU Variance	Method	${\mathrm{RMSE}}_{\mathrm{all}}$ (m)	${\mathrm{P95}}_{\mathrm{all}}$ (m)	${\mathrm{Max}}_{\mathrm{all}}$ (m)	${\mathrm{RMSE}}_{40–45}$ (m)	${\mathrm{P95}}_{40–45}$ (m)	${\mathrm{Max}}_{40–45}$ (m)
$10^{-3}$	Track-EKF	0.0419	0.1269	0.1972	0.1275	0.1622	0.1972
	Track-Gauss-AUKF	0.0108	0.0236	0.0483	0.0239	0.0352	0.0483
	Track-Adaptive KF	0.0414	0.1257	0.1714	0.1261	0.1495	0.1714
	Track-SW-FGO	0.0403	0.1246	0.1519	0.1230	0.1376	0.1519
	Proposed	0.0096	0.0185	0.0636	0.0103	0.0197	0.0636
$10^{-1}$	Track-EKF	0.0418	0.1267	0.1906	0.1271	0.1602	0.1906
	Track-Gauss-AUKF	0.0108	0.0235	0.0523	0.0238	0.0348	0.0523
	Track-Adaptive KF	0.0412	0.1262	0.1704	0.1256	0.1478	0.1704
	Track-SW-FGO	0.0527	0.1252	0.2309	0.1324	0.2033	0.2309
	Proposed	0.0099	0.0193	0.0993	0.0115	0.0220	0.0993

**Table 10:** RMSE (m) in NLoS scenarios under different LoS/NLoS interference models.

Scenario	Track-EKF	Track-Gauss-AUKF	Track-Adaptive KF	Track-SW-FGO	Proposed
AR(1)	0.130	0.024	0.129	0.124	**0.015**
Correlated-2link	0.386	0.344	0.382	0.374	0.012
Exponential	0.161	0.022	0.133	0.130	0.011
Gaussian	0.128	0.024	0.127	0.123	0.011
Lognormal	0.140	0.024	0.128	0.124	0.011
Mixture	0.142	0.024	0.125	0.120	0.013

**Table 11:** Hyperparameter sensitivity of the proposed method.

Sweep	Tested Values	${\mathrm{Min RMSE}}_{40–45}$ (m)	${\mathrm{Max RMSE}}_{40–45}$ (m)
$\lambda_{p}$	0.1, 0.3, 0.5, 1, 2	0.009	0.019
$N_{w}$	10, 15, 25, 35, 50	0.010	0.012
gate scale	0.6, 0.8, 1, 1.2, 1.5	0.010	0.041
$\lambda_{w},\beta$ grid	4 × 4	0.010	0.011

**Table 12:** Sensitivity to imperfect track-centerline knowledge.

Lateral Offset (m)	${\mathrm{RMSE}}_{\mathrm{all}}$ (m)	${\mathrm{RMSE}}_{40–45}$ (m)	${\mathrm{Max}}_{\mathrm{all}}$ (m)
0.00	0.009	0.011	0.035
0.02	0.022	0.022	0.042
0.05	0.051	0.051	0.065
0.10	0.101	0.101	0.108
0.20	0.201	0.201	0.206
0.50	0.500	0.501	0.509

**Table 13:** Online runtime.

Method	Mean Runtime (ms/step)	20 ms Budget/Mean Runtime
Track-EKF	0.107	186.8×
Track-Gauss-AUKF	0.264	75.8×
Track-Adaptive KF	0.087	230.7×
Track-SW-FGO	0.035	572.3×
Proposed	0.459	43.6×

**Table 14:** Ablation results under simultaneous multi-link NLoS conditions.

Variant	C1: Two NLoS-Affected Propagations (μ=1.0 m)		C2: Three NLoS-Affected Propagations (μ=2.5 m)	
	**${\mathrm{RMSE}}_{\mathrm{LoS}}$ (m)**	**${\mathrm{RMSE}}_{\mathrm{NLoS}}$ (m)**	**${\mathrm{RMSE}}_{\mathrm{LoS}}$ (m)**	**${\mathrm{RMSE}}_{\mathrm{NLoS}}$ (m)**
Full	0.011	0.377	0.012	0.305
w/o Consistency	0.012	0.413	0.013	0.305
w/o Hold	0.011	0.377	0.072	1.065
w/o Prior	0.018	0.470	1.832	24.863

**Table 15:** STAR-loc straight subsegments used for real-data feasibility testing.

Seg.	Dataset	Time (s)	*N*	Span (m)	Lat. RMS (m)	Range RMSE (m)
S1	zigzag_s4	34.0–46.0	241	4.320	0.025	0.187
S2	zigzag_s4	49.0–69.0	401	4.561	0.040	0.122
S3	zigzag_s3	28.1–38.1	201	4.142	0.053	0.177
S4	grid_s3	57.0–77.0	401	3.667	0.044	0.172
S5	apriltag_s3	83.0–98.0	301	3.757	0.111	0.266

**Table 16:** Average longitudinal localization error on the STAR-loc straight subsegments under the strict online calibrated-range protocol.

Method	RMSE (m)	MAE (m)	P95 (m)	Max (m)
Track-EKF	0.1266	0.0828	0.2977	0.4539
Track-Gauss-AUKF	0.8290	0.6737	1.5331	1.5907
Track-Adaptive KF	0.1610	0.1249	0.3249	0.4922
Track-SW-FGO	0.4099	0.3456	0.7276	0.8830
Proposed	0.0555	0.0451	0.1080	0.1507

**Table 17:** Ranging-error statistics.

Signal	RMSE (m)	Median (m)	P95 (m)	Max (m)
Unscreened range error	0.255	0.019	0.587	0.813
Screened propagation error	0.020	0.014	0.039	0.075

**Table 18:** Static localization summary in the track domain.

Method	Default RMSE (m)	Default P95 (m)	Max RMSE over μ (m)	Max RMSE over σ (m)
Track-1D-LS	0.128	0.167	0.252	0.174
Track-Robust LS	0.016	0.030	0.016	0.016
Proposed	0.086	0.111	0.168	0.116

## Data Availability

The STAR-loc dataset is publicly available from its original authors [27]. The core algorithm implementation and simulation data to reproduce the main findings of this study will be made available upon reasonable request.
